# Supplementary material for: Comparison of perceived stress and oral health status using perceived stress scale and DMFT index between healthy, undiagnosed hypertensive, and known hypertensive dental patients
Source: PLoS One. 2024 Oct 10;19(10):e0311645. doi: 10.1371/journal.pone.0311645 (PMC11469613; doi:10.1371/journal.pone.0311645)
Supplement: Attachment — Submitted filename: Response to Reviewers.docx [file pone.0311645.s002.docx]

**Date: 09 September 2024**

**To:**

**Hadi Ghasemi**

**The Academic Editor**

**and**

**The Reviewers**

**PLOS One**

Dear Academic Editor and Reviewers,

Subject: Authors’ response to reviewers’ comments and suggestions (PONE-D-24-30378 ‘Comparison of perceived stress and oral health status between healthy, suspected hypertensive, and known hypertensive dental patients’)

We have incorporated the suggestions into the revised manuscript. Thank you for your valuable comments and suggestions. A detailed response and the changes incorporated are given below:

Suggestions by Academic Editor:

Thank you for highlighting a very important aspect related to anonymity of the data. We have removed age column from the data.

**Comments 1-4:**

We thank the reviewers for their valuable time and for constructive feedback which has undoubtedly improved the revised manuscript.

**Comment 5:**

For Reviewer 1: We thank you for your review. For minor grammatical errors, these have been corrected on page 6, 7, 15, 17 and 18. Kindly let me know if there is anything specific that I might have missed.

For Reviewers 2,3,4: We are thankful for your valuable suggestions and comments working on which have undoubtedly improved the manuscript. We would highly appreciate if you could go through the revised manuscript and guide further if more corrections are needed. The following corrections are done:

| **Reviewer’s suggestion** | **Corrections** | **Line numbers** |
| --- | --- | --- |
| **Reviewer 1:** |  |  |
| Article is good in writing and structure. However, the relevance of the topic to the disease described is not clearly referred to and it is only the health behavior which is addressed. Suggestion is to edit the title accordingly | The title of the manuscript has been revised. Please let us know if the revised title is appropriate. | Page 1 and 2 |
| **Reviewer 2:** |  |  |
| 1. Small sample size and its focus on a single institution limit the generalizability of the findings. A larger, multi-center study would provide more robust and widely applicable results. | This has been added in the last paragraph of discussion as the limitation of this study. | 285-297 |
| 2. The use of purposive sampling rather than random sampling introduces potential bias, which could affect the study’s validity. Random sampling would have enhanced the representativeness of the sample. | It has been incorporated as study limitation | 288-291 |
| 3. Lack of control for confounding variables. The study does not account for potential confounding factors such as socioeconomic status, lifestyle factors (like diet and exercise), and other health conditions that could influence both stress and oral health. This omission weakens the conclusions drawn about the direct relationship between hypertension, stress, and oral health. | We have added table 6 for binary logistic regression analysis which accounts for the adjustment of possible confounders. Other limitations were mentioned in the last paragraph of discussion. Thank you for your valuable suggestions. | 156-158 |
| 4. Measurement Tools: While the DMFT index and PSS-10 are established tools, the study could benefit from a broader assessment of oral health and stress. Including measures like periodontal health or using more detailed psychological assessments might offer a more comprehensive understanding of the relationships explored. | It went beyond our study scope. However, we have included the suggestions in limitations and future recommendations | 294-297 |
| 5. Limited Discussion on Mechanisms: The discussion touches on possible links between hypertension and oral health but lacks a deeper exploration of the biological or behavioral mechanisms that might explain these relationships. Providing more context from existing literature would strengthen the study’s theoretical foundation. | We have added more details in discussion session about possible link between hypertension, stress and oral health. | 274-276 |
| 6. Short Study Duration: The study was conducted over just two months, which may not adequately capture the variations in stress and oral health that can occur over longer periods. Longitudinal studies would be better suited to observe the long-term effects of stress and hypertension on oral health. | We have included this as limitation. | 294-297 |
| 7. Absence of Longitudinal Data: The cross-sectional design limits the ability to draw causal inferences. Future research could benefit from a longitudinal approach to better establish the temporal relationship between hypertension, stress, and oral health deterioration. | We have included this as limitation and future recommendations | 294-297 |
| **Reviewer 3** |  |  |
| 1. The respected authors have used the term “suspected hypertension” which is not quit right. Based on the explained method, this includes patients with two times SBPs >130 or DBP>90. This would translate to undiagnosed HTN rather than suspects. This change would also mandate some revisions for the rationales stated for the rate of oral health status in this population in the discussion section. | We used suspected hypertension as we being dentists were not able to diagnose hypertension on our own and were in a position to refer such patients for proper diagnosis form a medical specialist. We have rephrased suspected hypertension with undiagnosed hypertension across the manuscript. | All |
| 2. I recommend using “patients with hypertension” instead of “hypertensive patients” across the manuscript as it has negative burden. | Thank you for your valuable input. It has been corrected across the manuscript. | All |
| 3. Please provide the exact purposive sampling method in line 85 as it can result in selection bias. | It is added. | 91-94 |
| 4. Lines 114-117 are repeated and can be removed. | It has been rephrased and repetition removed. | 119 |
| 5. The reference for validation of perceived stress, is based on age 19-30 years. As older population exist in the current study, I suggest elaborating it as A limitation of the study | We have added another reference for perceived stress in comparative age of 18-69 years. Thank you for highlighting this. | 129 |
| 6. In method section, were the continuous variables normally distributed? I’d suggest providing the appropriate statistical metrics for them. | It has been added that continuous variables were not normally distributed as per Shapiro Wilk’s test. Hence, non-parametric Kruskal Wallis test was used for comparison. | 149-152 |
| 7. Table 1 and 2 have the duplicate data due to the gender of the population in the study. | Sex from table 1 has been removed. | Table 1 page 7 |
| 8. In table 1 and result section, I’d highly recommend to add three extra columns reporting the characteristics based on the hypertension groups and incorporating them accordingly to the discussion section, as well. | Table has been modified and relevant discussion added. | Table 1 page 7, 216-225 |
| 9. Caution should be used when definitive association or equivalent terms are used in the result or discussion section, as the probable role of other possible co variates was not accounted for in this article. So, if possible, conducting a regression, with adjusting for confounding variables could significantly improve the conclusions of the study. | We have conducted binary regression analysis and have included it in results and discussion. | 198-205, Table 6, 255-266 |
| 10. In line 197, please provide more detail to compare your finding with other studies. | We have included another reference. | 225, 258-259 |
| Reviewer 4 |  |  |
| 1. Reference is needed for line 44. | Thank you for highlighting very important point. Reference is added. | 47 |
| 2. One or more references should be included in Page 3 line 60, page 7 line 214 since studies were mentioned. | References are added. | 64, 243 |

Thank you.

Yours sincerely,

Prof. Dr. Saira Atif (corresponding author)
